# Skin Cancer Risk, Sun-Protection Knowledge and Behavior in Athletes—A Narrative Review

**DOI:** 10.3390/cancers15133281

**Published:** 2023-06-22

**Authors:** Katarzyna Kliniec, Maciej Tota, Aleksandra Zalesińska, Magdalena Łyko, Alina Jankowska-Konsur

**Affiliations:** 1 Student Research Group of Experimental Dermatology, Department of Dermatology, Venereology and Allergology, Wroclaw Medical University, 50-368 Wroclaw, Poland; katarzyna.kliniec@gmail.com (K.K.); maciej.tota@student.umw.edu.pl (M.T.); aleksandrazalesinska@gmail.com (A.Z.); 2 Department of Dermatology, Venereology and Allergology, Wroclaw Medical University, 50-368 Wroclaw, Poland; alina.jankowska-konsur@umw.edu.pl

**Keywords:** photoprotection, athletes, skin cancer, prevention, sports, ultraviolet

## Abstract

**Simple Summary:**

Outdoor physical activities place athletes at risk of increased exposure to sunlight, which may result in sunburn, solar damage, and skin cancers. Therefore, awareness of proper sun protection methods and the risk of developing basal cell carcinoma, squamous cell carcinoma, and malignant melanoma in this group is crucial. In this review, we focus on a group of athletes and sports participants and summarize research concerning sun exposure during outdoor sports, the risk of developing skin cancer, and knowledge and behavior regarding photoprotection methods. Our analysis shows that outdoor sports participants do not protect themselves adequately from the sun. For this reason, it is essential to increase awareness of proper sun protection during outdoor sports practice to prevent skin cancers in the future.

**Abstract:**

Outdoor sports are associated with increased exposure to ultraviolet radiation, which may result in sunburn, solar damage, and skin cancers. Water and winter sports create additional adverse conditions, such as washing away sunscreen by water and reflection of UV rays by the water and snow. Sweating-increased skin photosensitivity and activity-induced immunosuppression are associated with a greater risk of developing skin cancers. In this review, we focus on a group of athletes and sports participants and analyze 62 articles concerning sun exposure during outdoor sports, the risk of developing skin cancer, and knowledge and behavior regarding photoprotection methods. Various practices have been linked to an increased risk of developing basal cell carcinoma (BCC), squamous cell carcinoma (SCC), malignant melanoma (MM), or UV-induced skin damage. Water sports and mountaineering increase the risk of BCC. Surfing and swimming are risk factors for SCC. Melanoma is more common in swimmers, surfers, and marathon runners. Photoprotection behaviors can reduce potential skin damage and skin cancers. Athletes’ knowledge about the risk of malignant lesions was satisfactory, but despite the risks, outdoor sports participants seem not to protect themselves from the sun adequately.

## 1. Introduction

There is no doubt that physical activity is beneficial to the body. It is associated with a reduced risk of several cancers, including of the colon, breast, and endometrium [1]. A specific type of physical activity is outdoor sports. Despite its positive effect on human health, outdoor recreation places athletes at risk of increased exposure to ultraviolet radiation (UVR), which may result in sunburn, solar damage, and a higher risk of skin cancer [2]. Sweating during sports can further increase the photosensitivity of sun-exposed skin [3]. In addition, activity-induced immunosuppression is considered another factor that may expose athletes to a higher risk of developing these types of cancers [1]. There are specific types of outdoor sports that involve additional negative factors associated with UV exposure. In water sports, the surrounding water reflects UV rays and washes photoprotective products off the skin, increasing the risk of sunburn [4]. During winter sports, snow reflects radiation and increases the skin’s UV exposure [5].

Solar exposure causes both chronic skin damage and sunburn [6]. The latter is caused by intense exposure, leading to an acute inflammatory response [7]. UV radiation is a primary risk factor for malignant melanoma (MM) and non-melanoma skin cancers (NMSCs) [5]. The incidence of both continues to rise worldwide [2,4,7,8]. MM and NMSCs are the most common cancers in Australia and New Zealand [2]. However, they are also among the most preventable cancers [4]. There are plenty of photoprotection methods, including sun-protective clothing with ultraviolet protection factors (UPFs), specific clothing (gloves, hats), and sunscreens [9]. There are also dedicated health promotion programs running educational campaigns to promote proper sun-related behaviors. 

We aimed to review the available literature concerning UV exposure during outdoor sports, the risk of developing skin cancer, and the application of photoprotection methods by athletes and sports participants based on their sun-protection knowledge and behavior. 

## 2. Materials and Methods

The literature search was performed between December 2022 and March 2023. The terms “outdoor sports”, “UV-radiation”, “athletes”, “sun exposure”, “outdoor physical activity”, “skin cancer”, “melanoma”, and “photoprotection” were searched in PubMed and Google Scholar. Only articles in English were accepted, and there were no restrictions on the publication date. The inclusion criterion was that the article address the following issues:

- consequences of sun exposure;
- skin cancer prevalence;
- association of outdoor sports with skin cancer incidence;
- available methods of photoprotection with particular emphasis on methods aimed at athletes;
- athletes’ sun-protection knowledge and behavior.

The exclusion criteria were (*n* = 91):

- no English-language version of the publication;
- full text not available;
- full text not available in English;
- publication not addressing the issues described in the inclusion criteria.

**Figure 1:**
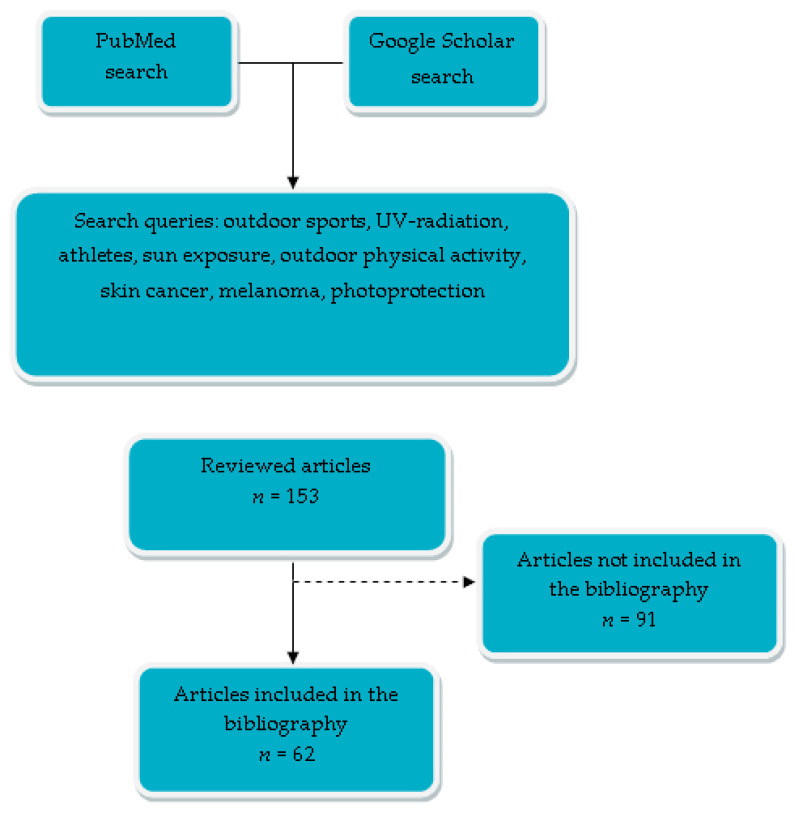
A flow chart of search strategy.

In the search, we identified 153 eligible articles. After title and abstract screening, 62 were assessed in full text and included in our manuscript. Figure 1 presents a flow chart of the search strategy. This article is a narrative review of previous studies of outdoor sports participants’ UV exposure, skin cancer risk, and sun-protection knowledge and behavior.

## 3. Results

### 3.1. Prevalence of Skin Cancer and UV-Induced Skin Lesions in Outdoor Athletes

Several studies have shown that various outdoor sports are risk factors for skin cancer [6,9,10]. This type of activity is associated with increased sun exposure and the occurrence of sunburn, which may promote melanoma and keratinocyte cancers [7,10,11]. Solar radiation causes immune system suppression and may trigger cancer development [5]. It is estimated that about two-thirds of melanomas and 90% of other skin cancers are related to overexposure to UV rays [12]. Furthermore, over-intensive training can cause tissue injury and thus induce immunosuppression [13]. In addition, sweating during sports may increase photosensitivity and exacerbate the harmful effects of UV exposure [5]. UV exposure may be caused by a change in the absorption spectrum of sunlight to shorter wavelengths as a result of hydration of the stratum corneum, thereby reducing reflection and diffusion [14]. This process presumably results in increased UVB transmission and higher UV sensitivity of moistened skin [3]. So far, however, no substance in sweat has been found to increase the skin’s sensitivity to sunlight. Further studies are needed to clarify whether any factor other than hydration of the stratum corneum could be responsible for this phenomenon.

Although, as mentioned above, a series of studies of outdoor sports have shown an increased incidence of skin cancers, reports on specific types of cancers have been inconsistent. Results have also differed depending on sports discipline.

There have been conflicting reports for keratinocyte cancers (basal cell carcinoma, BCC, and squamous cell carcinoma, SCC). 

Rosso et al. performed a multicenter, case-control study in seven southern European regions [15]. The study explored the link between sun exposure during different activities (outdoor sports, outdoor work, during holidays) and the occurrence of BCC and SCC. Researchers estimated the cumulative sun exposure time of the participants based on a standardized questionnaire. A comparison was performed among 1549 cases of BCC, 228 cases of SCC, and 1795 controls. The control group was selected through a random sampling process from the general population and was divided into subgroups based on the distribution of age and sex observed in the study group. There are no detailed data on the age and sex of the study population. No significant association was found between basal cell carcinoma (BCC) and outdoor sports, regardless of whether the duration of sun exposure was examined independently or adjusted for intensity and clothing. Moreover, the authors reported that outdoor sports did not incur an increased risk of SCC and may, in fact, be a protective factor. However, when focusing the analysis on sports activities characterized by high levels of sun exposure, a significant relationship was observed between basal cell carcinoma (BCC) and water sports (swimming, surfing, boating, and sailing). However, the relationship did not exhibit a distinct dose-response effect [15]. 

Dozier et al. conducted a screening for skin cancer in two populations—surfers and volunteers—who attended the annual skin cancer screening event [16]. The study took place on the Texas Gulf Coast of the USA. It included 49 surfers with a mean age of 27.9 years old and with a predominance of men. The control group consisted of 53 subjects with a mean age of 45.3 years old and with a predominance of women. In the mentioned cross-sectional study, the compared populations were heterogeneous in terms of gender and age. Surfers from the Texas Gulf Coast were screened for skin cancer and precancerous skin lesions [16]. The study authors grouped populations depending on age. The incidence rate of BCC was significantly higher in surfers aged 17 to 47 years old compared to the control group aged 18 to 47 years old. Forty-one percent of the surfers were diagnosed with actinic keratosis and 16 percent with BCC. This research is another study linking surfing to increased incidents of BCC. However, it should be emphasized that the compared groups differed in terms of gender. At the time of the study, epidemiological data indicated that men were more prone to developing skin cancers than women. Additionally, the age distribution significantly varied between the populations, with surfers exhibiting a considerably younger average age.

Climstein et al. conducted a cross-sectional study of the prevalence of skin cancer among Australian surfers and swimmers [17]. The study included 116 surfers and 55 swimmers. The authors reported that 41.4% of surfers and 36.4% of swimmers had a history of skin cancer. During the study, a total body skin examination revealed a significantly greater number of skin cancer cases in surfers (50%) compared to swimmers (27.3%). The most common finding was a precancerous condition, actinic keratosis, followed by BCC. There were seven cases of melanoma. The subjects exhibited higher odds ratios for BCC, SCC, and melanoma skin cancer compared to the general Australian population. Both surfers and swimmers commonly experienced skin cancers on the face. Additionally, a higher risk of melanoma was also observed in Australian swimmers and surfers than in the previously mentioned study [17].

The lifetime prevalence of skin cancer was examined among Australian recreational and competitive surfers in a 2016 study [18]. The study identified long-term surfing as the main risk factor, with a relatively higher risk observed in competitive surfers. The most common type of skin cancer among the surfers was basal cell carcinoma, and these cancers were found to occur more frequently on the face, back, and arms.

Lahmann et al., in a prospective study, analyzed the relationship between recreational and occupational outdoor activity and SCC [1]. They randomly selected 1621 residents of the Queensland township of Nambour, Australia, and observed them for 16 years. During the follow-up period, 98 men and 90 women were diagnosed with SCC. Their data suggested that there is a possible relationship between recreational activity time and the development of SCC in men. In women, a trend toward significance was observed between a higher level of occupational activity and a reduced incidence of SCC. However, both observations were only suggestive and not robust [1]. 

Another study conducted in Argentina included 101 melanoma cases and 246 controls [19]. The control group was matched for sex, age, and hospital. The authors of this case-control study found that outdoor sports are a risk factor for melanoma when associated with lifelong sun exposure, and the risk is proportional to the duration of exposure. This relationship was noted for recreational activity and was not confounded by other factors. The sports reported by subjects included football (the most common), tennis, swimming, cycling, rugby, and hiking.

Another case-control study investigated the risk factors for malignant melanoma specifically in marathon runners. The study included 210 subjects, with 166 men and 44 women, and the median age of the participants was 37 years old (ranging from 19 to 71 years). The control group was matched in terms of age and gender. The analysis of the study revealed that marathon runners exhibited an increased risk for both melanoma and non-melanoma skin cancer. Moreover, the presence of melanocytic nevi and actinic lentigines, which are known to be associated with the development of malignant melanoma, appeared to be more common among marathon runners [14]. 

Lawler et al., in a survey-based study, evaluated sun protection behaviors, sun exposure, and sunburn incidence in Australian young athletes during the previous sports season [20]. In the study, 237 participants aged 18–30 years old filled out questionnaires. The analyzed sport disciplines included surfing, tennis, field hockey, and soccer. The incidence of sunburn was estimated at 69 percent and was highest in surfers (93% of male and 84% of female surfers suffered sunburn in the previous season).

Another group worth mentioning is winter sports. The higher altitude, along with the reflection off of snow, in winter sports may increase the amount of UV radiation to which the skin is exposed [5]. Furthermore, people participate in these sports during periods of the highest ultraviolet radiation [12].

Lichte et al., in a study of 283 mountain guides, reported a significantly higher prevalence of sunburn and precancerous skin lesions in this occupational group [21]. All subjects were males with a median age of 41 years old. A control group comprised 309 age-matched men. Basal cell carcinoma (BCC) was diagnosed in 7.1% of them and squamous cell carcinoma (SCC) in 1.4%, and there was one case of malignant melanoma. No skin cancers were found among the 309 control subjects. The authors estimated that the risk of malignant melanoma is significantly higher in mountain guides compared to global statistics [21].

A 2015 study conducted in Germany on 503 patients diagnosed with melanoma and BCC suggested that mountaineering in childhood may increase the risk of sporadic BCC [8]. Surprisingly, the study found that the risk of melanoma was reduced compared to the control group or to the risk of BCC.

Price et al. found that 48% of their subjects had suffered sunburn while skiing or snowboarding, and 4% of them had a history of skin cancer [12].

These sports, of course, are not the only outdoor sports linked to skin cancer incidence. A study of 164 retired cricketers in Australia found that 38.4% of the subjects had a history of skin cancer [22]. The most common locations were the face (49 participants), followed by the arms, hands, neck, and chest.

Most outdoor physical activities increase exposure to UV radiation, which translates into an elevated risk of skin cancer. Particular attention should be paid to certain disciplines, especially water sports, which are associated with even greater exposure. As presented above, water sports seem to be addressed more frequently in the available literature on the association between outdoor physical activity and skin cancer. Individuals participating in water sports are exposed not only to UV radiation but also to the washing off of sunscreens. The water washes photoprotective products from the skin and reflects UV rays, which are another harmful factor [4]. Moreover, Australia offers favorable conditions for surfing, but for most parts of the year, Australia, Central America, and Africa experience higher cumulative UVR levels due to their proximity to the equator. The link between certain sports and specific types of skin cancer is not yet fully understood. However, our literature review revealed that basal cell carcinoma (BCC) is most frequently associated with water sports. This relationship can be attributed to several factors, including prolonged sun exposure, the nature of the exposure associated with BCC, and the aforementioned elements connected with water sports [23]. Limited information is available regarding squamous cell carcinoma (SCC) in sports based on the reviewed publications. Nevertheless, our analysis observed the occurrence of this cancer in water sports and marathon runners as well. SCC is also linked to prolonged sun exposure and cumulative exposure, with its development potentially involving the aforementioned factors related to water sports or extended exposure among marathon runners. Melanoma, apart from the aforementioned disciplines, has been associated in the literature with sports such as football, rugby, and tennis. In this case, the publications also do not provide an explanation for the association with this specific cancer. Melanoma is linked to intermittent, intense exposure, such as sunburn [23]. In the reviewed publications, study participants experienced sunburn, which may be one of the contributing factors.

Table 1 shows a summary of studies investigating the associations between sport disciplines and skin cancers and UV-induced skin lesions, as discussed in the literature.

### 3.2. Photoprotection in Athletes

Photoprotection behaviors can reduce potential skin damage in the forms of sunburn, erythema, pigmentary disorders, photoaging, precancerous lesions, and skin cancers [24,25]. 

Establishing awareness of photoprotection methods and their appropriate application in outdoor athletes is highly important given frequent ultraviolet radiation overexposure accompanied by profuse sweating [10].

Athletes practicing outdoor sports receive extreme doses of ultraviolet radiation associated with high sun exposure and, in alpine sports, an altitude-related increase in UV radiation and reflection from snow- and ice-covered surfaces [5]. Examples of such sports are skiing, mountaineering, cycling, running, triathlon, kitesurfing, windsurfing, beach handball, beach volleyball, and sailing. Summer sports are most often played during peak UV exposure hours (in the middle of the day) with uniforms that do not provide adequate sun protection [5]. During the Hawaiian Ironman Triathlon World Championships, the average UV exposure of three triathletes was approximately 20 standard erythema doses, with one dose equaling 100 J/m^2^ [5,26]. A minimal erythema dose (MED) is defined as the smallest amount of radiation exposure causing skin erythema or sunburn 24 h after exposure—approximately 200 to 300 doses of 100 J/m^2^ [5,26]. Cyclists in the Tour de Suisse were exposed to approximately eight times more than the minimal dose of UV needed to cause sunburn [27,28]. 

In winter sports (e.g., skiing, snowboarding), direct radiation occurs from the sun, often at higher altitudes, and from reflections off the snow and ice to exposed areas, such as the face and hands [5]. Rigel et al. demonstrated that skiers without sunscreen at 11,000 feet began to develop sunburn after only 6 min of UV exposure [28,29]. 

Due to frequent exposure to high levels of UV light, outdoor athletes are at risk for sunburn and the potential future development of skin cancer—especially athletes with lighter skin types [29]. Coaches and trainers may also be at increased risk because of similar exposure.

Increased perspiration associated with high sun exposure, heat, and exertion while practicing outdoor physical activities facilitates the erythema reaction and increases the risk of sunburn [2]. Moehrle et al. concluded that sweating exacerbates the effect of UVR exposure since it influences the hydration of the horny layer of the skin, resulting in a decrease in the reflection and dispersion of UV light [3]. 

To provide adequate sun protection, methods should be suitable for athletes’ individual needs considering their practicality for their discipline and training intensity, skin phototype, level of perspiration, and environmental factors, such as insolation, wind, temperature, and duration of sun exposure [30].

### 3.3. Photoprotection Methods

Photoprotective agents are divided into: (1) natural agents (occurring in nature, in the environment (ozone, clouds, fog, pollutants), and including the skin itself); (2) physical agents (clothes, hats, sunglasses), (3) sunscreens; and (4) antioxidants (found in the diet; they decrease the oxidative effects caused by exposure to ultraviolet radiation, such as vitamin E (tocopherol), vitamin C, beta-carotene, and flavonoids) [31,32,33].

Common photoprotection methods include seeking shade, avoiding sun exposure during peak daylight hours, wearing sun-protective clothing, applying sunscreen with a sun protection factor of 30 or greater, reducing exposure with long pants and long-sleeve shirts, and wearing hats and sunglasses [34,35]. These easy-to-follow rules implemented in everyday practice can contribute to reducing the risk of skin cancer.

Clothing is a highly effective photoprotective measure, especially for UVB [36,37,38]. A UPF of 40 to 50 has excellent UV protection, transmitting less than 2.6% of UV radiation, whereas a UPF of 25 to 39 has good UV protection [39,40]. Whether the fabric is wet or dry may increase or decrease UPF based on the type of fabric. Light-colored fabrics have decreased UPF compared to dark-colored fabrics [24]. There are commercially available laundry detergents with additives that absorb UVR and increase UPF [31,41]. Athletes, when choosing sportswear, should consider the above. Overall, clothing provides a balanced amount of protection against both UVA and UVB, and a loose-fitting, colored fabric is the best form of photoprotection [32].

Regarding the individuals involved in water sports, the water resistance of sunscreen products is highly important [42]. Resistant or highly water-resistant sunscreens should be reapplied after 40 min of swimming and 3 h of sweating [43]. Athletes exposed to UV radiation should be encouraged to use a broad-spectrum water-resistant sunscreen or a very water-resistant sunscreen with an SPF of at least 30-50 [29]. A high-SPF lip balm is also recommended. Sweating, friction, and water immersion will decrease the effectiveness of sunscreens if not properly reapplied. Alcohol-based sunscreen sprays, gels, and lotions are not as heavy or greasy as creams and are better tolerated by athletes. Hamant and Adams surveyed collegiate athletes and found that 85% reported no sunscreen use in the previous week, and only 6% used sunscreen at least three days of the week during the study [44]. 

Raising the awareness and application of photoprotection methods among outdoor athletes is essential for their skin health and protection against sun damage.

### 3.4. Athletes’ Awareness and Application of Possible Photoprotection Methods

De Castro-Maqueda et al. examined the attitudes among male elite kite surfers and found that 84.7% of respondents had at least one sunburn in the previous season. Sports participants were administered a questionnaire to reflect on their knowledge about sun exposure and skin cancer; 86.6% of participants did not believe that using sunscreen cream is the most appropriate way to protect themselves from the sun and prevent skin cancer. Consequently, 79.2% applied SPF > 15, 37.5% applied SPF > 50 sunscreen cream, and only 4.2% reapplied it every hour. The authors underscored that this misunderstanding may be a reason for high rates of sunburn [45]. The results showed that the study group did not equate the higher risk of SCC with sunburns. Moreover, knowledge about the indispensable reapplication of sunscreen cream after 30 min should be spread, especially in those playing water sports.

Another study targeted beach handball players. A group of 121 athletes divided into two groups based on age (university students and younger players) was administered a questionnaire. The study showed that younger players made less use of sunscreen cream (43.7% vs. 50.7%). Consequently, they were more prone to sunburn (81.25% vs. 73.97%). Interestingly, only 47.9% of university students reapplied sunscreen cream compared to 65% of younger players. The authors emphasized that reapplication is important and should be undertaken after 30 min to ensure appropriate protection [46].

Athletes that underwent organ transplantation were also examined. In this study, the authors showed that therapy with cyclosporine led to significantly fewer sunburns in the previous year compared to other immunosuppressants (tacrolimus, mycophenolate mofetil/mycophenolic acid, corticosteroids, sirolimus, azathioprine, and everolimus). Nevertheless, that information should be confirmed by future studies due to the limited patient population on cyclosporine. The sunburns were less often noted than in other study groups (28.9% in the previous season). These incidents were significantly more common in younger transplant athletes. Moreover, lower education status correlated with more sunburns [47]. The study revealed a relatively high awareness of photoprotection in transplant athletes.

The most popular photoprotection technique among runners was wearing sunglasses. Regarding sunscreen cream, 49% claimed that they forgot to apply it, and 17.3% felt uncomfortable wearing it. The authors indicated that sun protection was more prevalent in women, older runners, those who ran fewer miles per week, those with lower BMI, and those who experienced skin cancer [48].

Water sports athletes, such as sailors, surfers, or windsurfers, are highly exposed to ultraviolet radiation ranging from 4 h 51 min/day in sailors to 3 h 53 min/day in windsurfers. De Castro-Maqueda et al. confirmed that older participants have more adequate photoprotection habits than younger athletes. In the previous season, 76.7% of participants suffered at least one sunburn. The highest rate of sunburn was found in windsurfers (86.1% men, 83.3% women) [4].

Gutiérrez-Manzanedo et al. examined the ultraviolet exposure of competitors during a Tokyo Olympic Sailing Regatta Test Event. Athletes’ habits were analyzed during three competition days. Surprisingly, 46.2% of participants experienced at least one sunburn during a relatively short period, and 23.1% had at least three sunburns. Only 11.8% of competitors used sunscreen cream frequently [49].

A study conducted by Wysong et al. on collegiate athletes confirmed that the most common reason for not applying sunscreen cream was forgetfulness (63%). Regrettably, 73% of participants had never or rarely discussed sun exposure and sunscreen use with a coach or athletic administrator. Higher sunscreen use was found in female students, those who experienced more sunburns in the previous year, those who believed they were at risk for skin cancer as an athlete, those who knew someone with skin cancer, those who had skin examinations, and those who were worried about wrinkles, sunburns, or skin cancer [50].

Among athletes competing in the Croatian Olympic and Super Sprint triathlon, 27% reported frequent use of sunscreen cream, while only 3% always used sunscreen. One in five participants (20%) had never used sunscreen. Hence, 26% of athletes had severe sunburn with blisters in the past [51].

Wearing long-sleeved shirts and trousers was the most popular photoprotection practice among skaters (65.9%). Only 18.7% used sunscreen cream frequently. More than half of the participants (56.8%) experienced at least one sunburn in the previous season. The study has brought educational benefits: 58% of participants learned to protect themselves from the sun, and 57% claimed to start protecting themselves [52].

Weikert et al. investigated golfers’ attitudes and photoprotection techniques. Among the participants, 59% reported always or often wearing sunscreen, 65% wore long sleeves, and 68% wore a hat while golfing. Despite these reporters, 70% of golfers had at least one sunburn in the previous year [53].

Of 2445 runners, only 23.5% had adequate sun exposure and protection behaviors. More women presented proper photoprotection habits (33% vs. 17%). Despite this finding, the sunburn rate was relatively low, ranging from 6% to 10%. Runners younger than 45 years old were more likely to experience sunburn [54].

Another study of runners indicated that risk factors for sunburn are younger age, low Fitzpatrick skin type (I and II), and running 3+ hours a day. Conversely, protective factors are the use of sunscreen and seeking shade. In the previous year, 45.1% of participants reported sunburn. The most popular photoprotection techniques were sunglasses (74.7%), sunscreen (61.9%), wearing a hat (52.2%), and wearing other protective clothing (7.4%) [55].

Cyclists are another group exposed to high doses of UV radiation. The sunburn rate was 45.6%, with 53.8% for those <40 years old and 39.7% for those 40+ years old [56]. The relationship with age is similar to that observed in runners [54]. Risk factors for sunburn include younger cyclists (<40 years old), those with skin type I or II, those exposed to the sun at least three hours per week, and those who (almost) never use sunscreen [56].

Cohen et al. found that female collegiate athletes are more likely than male athletes to reapply sunscreen after prolonged sun exposure (58.3% vs. 37.5%) [57]. Forgetfulness was the main reason for not using sunscreen cream, as reported in other articles [48,50].

Despite relatively high rates of photoprotection in paralympic sailors, 76.8% of them reported sunburn in the previous season. Among sailors, 83.9% reported using sunscreen, but only 16.1% reapplied it every one or two hours. Sunglasses were the most common photoprotection method (85.7%) [58].

The results obtained by Christoph et al. showed that older (between 35 and 54 years of age), fair-skinned (type I and II), and female runners have higher skin cancer awareness and better protection behaviors. Among the runners, 34.4% of participants reported at least one sunburn before adulthood, and 3.9% had a history of skin cancer. Almost 15% had a positive family history of skin cancer (Table 2). Those with a positive personal history of skin cancer had a higher photoprotection score. Conversely, a positive family history of skin cancer did not correlate with a higher photoprotection score [59].

A summary of studies of athletes’ awareness and application of possible photoprotection methods is presented in Table 2. 

### 3.5. Photoprotection and Skin Cancer Awareness Campaigns Targeting Athletes

In recent years, several campaigns have been initiated to promote photoprotection and skin cancer awareness among athletes. As part of these efforts, a significant study was conducted in 1992 involving 49 surfers who participated in the Texas Gulf Coast competition [16]. Any participants found to have suspicious lesions were advised to seek medical attention. The results of their examinations were then compared with those of a control group. The study revealed a higher prevalence of basal cell carcinoma (BCC) among surfers; thus, the authors reported the validity of targeting this specific group with the campaign.

Walkosz et al. conducted an evaluation to assess the effectiveness of the Go Sun Smart program in North America [60]. The campaign involved 26 ski resorts that were randomly assigned to either the control or the intervention group, ensuring that any observed differences in outcomes could be attributed to the Go Sun Smart program. The study interviewed 357 parents of children enrolled in ski and snowboard schools at these resorts, assessing their photoprotection behaviors and exposure to the campaign. The control group comprised parents from resorts without the program. The findings showed that a larger proportion of children in the intervention group used sunscreen compared to the control group, but this difference was not statistically significant in most regions. However, it was found to be significant in the Northwest region. Furthermore, parents from the intervention group were significantly more likely to report having seen the Go Sun Smart posters, but no significant differences were found for verbal information provided by resort employees regarding sun protection. Based on these results, the authors suggest that winter sports resorts serve as a potentially effective venue for implementing sun protection education campaigns [60].

Another study, conducted by the same authors, focused on the adult population and involved 6516 adult guests at 26 ski areas in the USA and Canada [61]. The intervention campaign utilized various strategies, such as informational posters, brochures, and signs placed at the bases of chairlifts and on chairlift poles. Additionally, programs were implemented for employees to encourage them to educate guests about the risks of overexposure to the sun.

Participants were interviewed to gather information about their photoprotection behaviors, incidences of sunburn, recall of sun-protection information from the campaign, and the association between exposure to the campaign and sun protection practices. The findings revealed a significant difference between the intervention and control resorts in terms of the participants’ recall of photoprotection information. Moreover, the intervention group demonstrated higher usage of photoprotection measures.

Based on these results, the authors concluded that the signs used in the campaign had the highest impact in terms of exposure to sun-protection information, and the overall campaign was effective for guests who encountered and remembered the messaging.

In another study, the impact of the informational campaign “SUNSPORT” on student-athletes’ photoprotection beliefs and behaviors was evaluated [62]. The campaign involved 846 participants who attended education sessions conducted by a dermatologist during their annual skin examinations. Presentations and materials were provided to enhance their understanding of the topic. To evaluate the effectiveness of the SUNSPORT intervention, participants were interviewed both before and after the campaign. The findings revealed a statistically significant increase in the number of student-athletes who used sunscreen at least four times per week. Additionally, there was an improvement in their awareness of the risks associated with skin cancer and an increase in the frequency of discussions with their coaches about sun safety.

These studies emphasize the impact of tailored interventions in increasing awareness, changing behaviors, and reducing risks associated with sun exposure.

## 4. Conclusions

Outdoor physical activities are associated with increased solar exposure and a higher risk of developing skin cancer. Certain sports, such as water or winter sports, create additional adverse conditions. Activity-induced immunosuppression and increased skin photosensitivity due to sweating also work against athletes. Despite the risks, outdoor sports participants seem to not protect themselves from the sun adequately. The most prevalent photoprotection methods were wearing sunglasses, sunscreen cream, long-sleeved T-shirts and trousers, and hats or caps; avoiding the midday sun; and staying in the shade. Although knowledge about the risk of malignant skin lesions was satisfactory, the sunburn rates were very high, up to 84,7% in kite surfers. Sunscreen use in particular was underappreciated, with forgetfulness as the main reason for not applying it. Not least of all, the reapplication of sunscreen cream was very rare. 

## 5. Perspectives

Inadequate sun protection among athletes is an urgent issue. Federations and other sports organizations could implement awareness-raising campaigns addressing sun exposure practices and photoprotection behaviors. We suggest organizing repeated training sessions on proper photoprotection habits. Sunscreen cream use and appropriate reapplication timing should be strongly encouraged. Famous athletes and other influential people could be involved to enhance the attractiveness of campaigns.

## Figures and Tables

**Table 1:** Summary of studies investigating the association between sport discipline and skin cancers and UV-induced skin lesions.

References and Year of Publication	Study Design	Number of Group	Mean Age of Participants	Geographical Location	Type of Cancer, UV-Induced Skin Lesions, Sunburns	Disciplines at Risk
Rosso et al. [15], 1996	The study recruited cases of BCC and SCC from population-based cancer registries and hospitals in seven southern European regions. A control group was also recruited from the same regions. Sun exposure was assessed using a structured questionnaire that collected information on the duration, season, hours of exposure, and clothing during outdoor activities.	1549 BCC cases, 228 SCC cases, 1795 controls	ND	Southern Europe (Turin, Italy; Trento, Italy; Ragusa, Italy; Villejuif, France; Creteil, France; Besan, France; Murcia, Spain; Granada, Spain)	BCC	Swimming, sailing, surfing
Climstein et al. [17], 2022	Participants in this cross-sectional study were recruited through notices sent to local general practitioners, surf and swim clubs, and the local media. During the study, a questionnaire and full-body screening for skin cancer were conducted.	94 men, 77 women	43.9	Southeast Queensland and Northern New South Wales, Australia	BCC	Swimming, surfing
					SCC	
					Melanoma	
Climstein et al. [18], 2016	This cross-sectional observational study used a customizable, commercially available survey directed at Australian surfers to estimate the lifetime incidence of skin cancer.	1231 men, 117 women	35.84	Australia	BCC	Surfing
Dozier et al. [16], 1997	The researchers performed a cross-sectional study involving surfers from the Texas Gulf Coast who were interviewed and screened for skin cancer by dermatologists during surfing competitions.	44 men, 5 women	29.7	Galveston, Texas, USA	BCC	Surfing
Kaskel et al. [8], 2015	Participants diagnosed with cutaneous melanoma or sporadic basal cell carcinoma within the previous six months to five years were included in the study. Control subjects without any cancer were also included. The study involved interviews, examinations, and standardized questionnaires to compare UV exposure behavior and assess sun sensitivity.	Melanoma patients: 142 men, 149 women; BCC patients: 102 men, 110 women; Controls: 165 men, 164 women	Melanoma patients: Median age at primary diagnosis—55 BCC patients: Median age at primary diagnosis—69 Controls: 57	Ulm, Germany; Dresden, Germany	BCC	Mountaineering
Loria et al. [19], 2001	The study included 101 cases of verified melanoma and 246 controls. Participants were interviewed about demographic and constitutional factors and solar exposure history.	Melanoma cases: 55 men and 46 women Controls: 246	ND	Buenos Aires, Argentina	Melanoma	Football, rugby, tennis, swimming, cycling, hiking
Ambros-Rudolph et al. [14], 2006	The study participants were recruited during the Graz marathon, while the control subjects were recruited during a skin cancer screening campaign in Graz and Styria. The study specifically included white residents of Styria. The participants were interviewed about risk factors for malignant melanoma and their training habits. Additionally, a full-body skin examination was conducted by an experienced dermatologist for all participants undergoing skin cancer screening.	Marathon runners: 166 men, 44 women; Controls: 210	Marathon runners: 37 Controls: ND	Styria, Austria; Graz, Austria	Melanoma, non-melanoma skin cancer, melanocytic nevi actinic lentigines/solar lentigo	Marathon runners
Lichte et al. [21], 2010	The study included 283 male mountain guides and 309 control subjects. They were interviewed about personal and occupational data, UV exposure, photosensitivity, sunburn, sun protection measures, and history of previous skin cancer. They were examined for UV-related skin damage and skin cancer; suspicious lesions were biopsied.	592 men	41	Germany, Switzerland, Austria	Melanoma, solar keratoses/actinic keratosis, Sunburn	Mountain guides
Noble-Jerks et al. [22], 2006	The study was based on a self-report questionnaire aimed at retired cricketers. Questions included personal information, playing, employment and medical history, the impact of injuries, training, current physical activity, use of sun protection, and history of skin cancer.	164 men	45.2	Australia	Skin cancer (unspecified)	Cricket
Price et al. [12], 2003	Skiers and snowboarders were interviewed via a questionnaire about personal information, sun protection behavior, skin type, skin cancer history, and awareness of educational messages.	139 men, 87 women	no data	Queenstown, New Zealand	Sunburn	Skiing, Snowboarding
Lawler et al. [20], 2007	Field hockey players, soccer players, tennis players, and surfers were asked to complete a self-administered questionnaire concerning demographic information and sun protection habits during sports. The study compared sun protection behaviors by discipline and sex.	97 men, 140 women	23.2	Australia	Sunburn	Surfing, tennis, field hockey, soccer

ND—no data; BCC—basal cell carcinoma; SCC—squamous cell carcinoma.

**Table 2:** Summary of studies on athletes’ awareness and application of possible photoprotection methods.

References	Year of Publication	No. of Group	Discipline	Mean Time of Exposure	Sunburns	Awareness/Attitude	Application/Methods of Photoprotection
De Castro-Maqueda et al. [45]	2022	72	kitesurfing	10.93 years; 13.93 h per week	84.7% at least 1 sunburn; 43.1% 3 sunburns	79.2% worried about skin cancer	79.2% SPF > 15 sunscreen cream; 37.5% SPF > 50 sunscreen cream; only 4.2% reapplied the cream every hour; 40.3% reapplied it after 3 h; 62.5% wear sunglasses; 20.8% wear a long-sleeved shirt and long trousers 50% wear a cap or hat 6.9% avoid the sun (12:00–14:00)
De Castro-Maqueda et al. [46]	2019	121	beach handball	57.5% less than 2 h of sun exposure (Monday to Friday); 31.5% during the weekend	76.9% at least 1 sunburn	94.5% of university students (US) and 64.6% of younger students (YS) believe that sunscreen cream prevents aging of the skin; 97.3% of the US and 85.4% of the YS believe that the sun is the leading cause of skin cancer	68.5% of university students and 66.7% of younger athletes used sunscreen cream (SPF ≥ 30); 52.1% of students and 35.4% of younger players did not reapply it
Navarrete-De Gálvez et al. [47]	2021	170	athletes who underwent organ transplantation	61.3% of athletes had been playing sports for >15 years; 79.5% spent >1–2 h a day outdoors	28.9%	72.8% considered sunbathing harmful to their health; 78.3% do not like sunbathing; 87% believe that it is worthwhile to use sunscreen cream; 24.2% were not worried about sunburn	68.9% sunscreen cream; 67.3% sunglasses; 48.8% hat; 15.7% long-sleeved shirt and long trousers; 42.2% avoid midday sun (12:00–16:00); 58.2% shadow/umbrella
Tenforde et al. [48]	2021	697	runners	ran 37.4 ± 17.7 miles per week	ND	39.2% fear skin cancer; 15.8% fear skin aging; 49% forget to use sunscreen; 17% feel uncomfortable with sunscreen;	45% sunglasses; 43% avoid midday sun exposure (10:00–14:00); 42% sunscreen on face; 40% wear a hat; 10% run in shade; 7% wear long sleeves
De Castro-Maqueda et al. [4]	2021	240	water sports	sailors—4 h 51 min per day; surfers—4 h 35 min per day; windsurfers—3 h 53 min per day	76.7% 1 sunburn; 27.5% 3+ sunburns	ND	ND
Gutiérrez-Manzanedo et al. [49]	2022	13	sailors	mean daily personal UV exposure received was 761.0 ± 263.6 J/m^2^, 3.0 ± 1.1 minimal erythemal dose and 7.6 ± 2.6 standard erythemal dose (in 3 competition days)	46.2% 1 sunburn; 23.1% 2 sunburns (in 3 competition days)	ND	94.2% wear T-shirts; 50.2% shade; 44% wear a hat/cap; 26.1% sunglasses; 11.8% sunscreen
Wysong et al. [50]	2012	290	collegiate athletes	4 h per day; 10 months per year of training outdoors.	84% 1 sunburn; 28% 4+ sunburns	96% agreed that sunscreen helps prevent skin cancer; 63% forget to use sunscreen	more than 50% never used sunscreen; 75% used sunscreen 3 or fewer days/week
Buljan et al. [51]	2020	95	triathlon athletes	30% of participants spent 4 to 6 h per week outdoors; 21% spent more than 10 h outdoors per week	26% reported previously having severe sunburns with blisters	more than 90% of participants stated it was essential to use sunscreen; however, almost 50% rarely used sunscreen while training, 27% frequently used sunscreen, while only 3% always used sunscreen.	20% of participants never used sunscreen
Fernández-Moranoet al. [52]	2017	102	skaters	91.6% practiced outdoor sports more than 90 days per year; 6.1% spent this long at the beach; 52.1% practiced outdoor sports for 5 or more hours a day; 43.3% who spent comparable periods at the beach	56,8% at least one sunburn	58% of participants learned to protect themselves from the sun; 57% claimed to start protecting themselves	20% sunglasses; 33% wear a hat/cap; 65.9% long-sleeves; 18.7% sunscreen SPF > 15; 23.3% avoid peak sun
Weikert et al. [53]	2021	347	golfers	more than 7.5 h per week	70% at least one sunburn	61% believed that golf increased their risk for skin cancer a little or much; 39% of golfers did not believe that golfing increased their risk for skin cancer, including 14% who believed golfing lowers their risk for skin cancer	59% sunscreen; 65% long-sleeves; 68% wear a hat/cap; 33% shade/umbrella
Duarte et al. [54]	2018	2445	runners	51% spent 4+ hours training outdoors per week	10% < 45 y.o.; 6% > 45 y.o.	90% know that melanoma is a malignant lesion; 37% know that basal cell carcinoma is malignant	30% sunglasses; 17% wear a hat/cap; 12% sunscreen cream; 4% long-sleeves
García-Malinis et al. [55]	2020	657	runners	ND	45.1%	ND	74.7% sunglasses; 61.9% sunscreen (SPF ≥ 15) 52.2% wear a hat/cap; 7.4% other protective clothing
Molinero et al. [56]	2020	1018	cyclists	67.3% more than 90 days per year while cycling; 59.3% trained for more than 3 h per training session	45.6%	61.0% did not examine their skin regularly	95.5% wear a hat/cap; 92.8% sunglasses; 39.2%sunscreen cream
Cohen et al. [57]	2006	1006	collegiate athletes	16.1 h per week	20% sunburn with blisters	95.1% agreed that sun exposure could cause skin cancer; 91.1% of athletes agreed that sun exposure could cause premature aging of the skin; 30.5% of athletes agreed that a white cotton T-shirt adequately protects the skin underneath from sunburn	37.7% sunscreen cream; 34.7% wear a hat/cap; 27.9% long-sleeved shirts; 38.8% long-sleeved trousers
Gutiérrez-Manzanedo et al. [58]	2021	56	paralympic sailors	8 h per week	76.8%	82.1% worried about skin cancer from the sun; excessive sun tanning attitudes (42.9% liked sunbathing, and 57.1% said sunbathing makes them feel well)	85.7% sunglasses; 83.9% sunscreen cream; 33.9% do not reapply it, and 16.1% reapply it every 1 or 2 h; 75% wear a hat/cap; 28.6% avoid the midday sun
Christoph et al. [59]	2016	970	runners	ND	34.4% experienced severe sunburn before adulthood	ND	ND

ND—no data; SPF—sun protection factor; UV—ultraviolet, y.o.—years old.

## References

[1] Lahmann P.H., Russell A., Green A.C. (2011). Prospective Study of Physical Activity and Risk of Squamous Cell Carcinoma of the Skin. BMC Cancer.

[2] Snyder A., Valdebran M., Terrero D., Amber K.T., Kelly K.M. (2020). Solar Ultraviolet Exposure in Individuals Who Perform Outdoor Sport Activities. Sport. Med.-Open.

[3] Moehrle M., Koehle W., Dietz K., Lischka G. (2000). Reduction of Minimal Erythema Dose by Sweating. Photodermatol. Photoimmunol. Photomed..

[4] De Castro-Maqueda G., Gutierrez-Manzanedo J.V., Lagares-Franco C., de Troya-Martin M. (2021). Sun Exposure during Water Sports: Do Elite Athletes Adequately Protect Their Skin against Skin Cancer?. Int. J. Environ. Res. Public Health.

[5] Moehrle M. (2008). Outdoor Sports and Skin Cancer. Clin. Dermatol..

[6] Gilaberte Y., Trullàs C., Granger C., de Troya-Martín M. (2022). Photoprotection in Outdoor Sports: A Review of the Literature and Recommendations to Reduce Risk Among Athletes. Dermatol. Ther..

[7] Wu S., Cho E., Li W.Q., Weinstock M.A., Han J., Qureshi A.A. (2016). History of Severe Sunburn and Risk of Skin Cancer Among Women and Men in 2 Prospective Cohort Studies. Am. J. Epidemiol..

[8] Kaskel P., Lange U., Sander S., Huber M.A., Utikal J., Leiter U., Krähn G., Meurer M., Kron M. (2015). Ultraviolet Exposure and Risk of Melanoma and Basal Cell Carcinoma in Ulm and Dresden, Germany. J. Eur. Acad. Dermatol. Venereol..

[9] Morton S.K., Harrison S.L. (2022). Slip, Slop, Slap, Slide, Seek and Sport: A Systematic Scoping Review of Sun Protection in Sport in Australasia. Curr. Oncol..

[10] Wolf S.T., Kenney L.E., Kenney W.L. (2020). Ultraviolet Radiation Exposure, Risk, and Protection in Military and Outdoor Athletes. Curr. Sports Med. Rep..

[11] Leiter U., Eigentler T., Garbe C. (2014). Epidemiology of skin cancer. Adv. Exp. Med. Biol..

[12] Price J., Ness A., Leary S., Kennedy C. (2006). Sun-Safety Behaviors of Skiers and Snowboarders on the South Island of New Zealand. J. Cosmet. Dermatol..

[13] Smith L.L. (2003). Overtraining, Excessive Exercise, and Altered Immunity: Is This a T Helper-1 versus T Helper-2 Lymphocyte Response?. Sports Med..

[14] Ambros-Rudolph C.M., Hofmann-Wellenhof R., Richtig E., Müller-Fürstner M., Soyer H.P., Kerl H. (2006). Malignant Melanoma in Marathon Runners. Arch. Dermatol..

[15] Rosso S., Zanetti R., Martinez C., Tormo M.J., Schraub S., Sancho-Garnier H., Franceschi S., Gafà L., Perea E., Navarro C. (1996). The Multicentre South European Study “Helios”. II: Different Sun Exposure Patterns in the Aetiology of Basal Cell and Squamous Cell Carcinomas of the Skin. Br. J. Cancer.

[16] Dozier S., Wagner R.F., Black S.A., Terracina J. (1997). Beachfront Screening for Skin Cancer in Texas Gulf Coast Surfers. South. Med. J..

[17] Climstein M., Doyle B., Stapelberg M., Rosic N., Hertess I., Furness J., Simas V., Walsh J. (2022). Point Prevalence of Non-Melanoma and Melanoma Skin Cancers in Australian Surfers and Swimmers in Southeast Queensland and Northern New South Wales. PeerJ.

[18] Climstein M., Furness J., Hing W., Walsh J. (2016). Lifetime Prevalence of Non-Melanoma and Melanoma Skin Cancer in Australian Recreational and Competitive Surfers. Photodermatol. Photoimmunol. Photomed..

[19] Loria D., Matos E. (2001). Risk Factors for Cutaneous Melanoma: A Case–Control Study in Argentina. Int. J. Dermatol..

[20] Lawler S., Spathonis K., Eakin E., Gallois C., Leslie E., Owen N. (2007). Sun Exposure and Sun Protection Behaviours among Young Adult Sport Competitors. Aust. N. Z. J. Public Health.

[21] Lichte V., Dennenmoser B., Dietz K., Häfner H.M., Schlagenhauff B., Garbe C., Fischer J., Moehrle M. (2010). Professional Risk for Skin Cancer Development in Male Mountain Guides--a Cross-Sectional Study. J. Eur. Acad. Dermatol. Venereol..

[22] Noble-Jerks J., Weatherby R.P., Meir R. (2006). Self-Reported Skin Cancer Protection Strategies and Location of Skin Cancer in Retired Cricketers: A Case Study from Membership of the Emu Cricket Club. J. Sci. Med. Sport.

[23] Savoye I., Olsen C.M., Whiteman D.C., Bijon A., Wald L., Dartois L., Clavel-Chapelon F., Boutron-Ruault M.C., Kvaskoff M. (2018). Patterns of Ultraviolet Radiation Exposure and Skin Cancer Risk: The E3N-SunExp Study. J. Epidemiol..

[24] Gabros S., Nessel T.A., Zito P.M. (2023). Sunscreens and Photoprotection.

[25] Taylor C.R., Stern R.S., Leyden J.J., Gilchrest B.A. (1990). Photoaging/Photodamage and Photoprotection. J. Am. Acad. Dermatol..

[26] Moehrle M. (2001). Ultraviolet Exposure in the Ironman Triathlon. Med. Sci. Sports Exerc..

[27] Moehrle M., Heinrich L., Schmid A., Garbe C. (2000). Extreme UV Exposure of Professional Cyclists. Dermatology.

[28] Rigel D.S., Rigel E.G., Rigel A.C. (1999). Effects of Altitude and Latitude on Ambient UVB Radiation. J. Am. Acad. Dermatol..

[29] Adams B.B. (2002). Dermatologic Disorders of the Athlete. Sports Med..

[30] Pinheiro M.C., Reis-Mansur P., Gonçalves Da Luz B., Pereira E., Santos D. (2023). Consumer Behavior, Skin Phototype, Sunscreens, and Tools for Photoprotection: A Review. Cosmetics.

[31] Kullavanijaya P., Lim H.W. (2005). Photoprotection. J. Am. Acad. Dermatol..

[32] Rai R., Shanmuga S., Srinivas C.R. (2012). Update on Photoprotection. Indian J. Dermatol..

[33] Pinnell S.R., Yang H., Omar M., Riviere N.M., DeBuys H.V., Walker L.C., Wang Y., Levine M. (2001). Topical L-Ascorbic Acid: Percutaneous Absorption Studies. Dermatol. Surg..

[34] Tsai J., Chien A.L. (2022). Photoprotection for Skin of Color. Am. J. Clin. Dermatol..

[35] Jinna S., Adams B.B. (2013). Ultraviolet Radiation and the Athlete: Risk, Sun Safety, and Barriers to Implementation of Protective Strategies. Sports Med..

[36] Georgouras K.E., Stanford D.G., Pailthorpe M.T. (1997). Sun Protective Clothing in Australia and the Australian/New Zealand Standard: An Overview. Australas J. Dermatol..

[37] Gambichler T., Altmeyer P., Hoffmann K. (2002). Role of Clothes in Sun Protection. Recent Results Cancer Res..

[38] Sk M.S., Akram W., Mia R., Fang J., Kabir S.M.M. (2022). Fabrication of UV-Protective Polyester Fabric with Polysorbate 20 Incorporating Fluorescent Color. Polymers.

[39] Lim H.W., Goldsmith L.A., Katz S.I., Gilchrest B.A., Paller A.S., Leffell D.J., Wolff K. (2012). Chapter 223. Photoprotection. Fitzpatrick’s Dermatology in General Medicine, 8e.

[40] Gambichler T., Rotterdam S., Altmeyer P., Hoffmann K. (2001). Protection against Ultraviolet Radiation by Commercial Summer Clothing: Need for Standardised Testing and Labelling. BMC Dermatol..

[41] Gontijo G.T., Pugliesi M.C.C., Araújo F.M. (2009). Photoprotection. Surg. Cosmet. Dermatol..

[42] Puccetti G., Fares H. (2014). A New Approach for Evaluating the Water Resistance of Sunscreens on Consumers: Tap Water vs. Salt Water vs. Chlorine Water. Int. J. Cosmet. Sci..

[43] Hexsel C.L., Bangert S.D., Hebert A.A., Lim H.W. (2008). Current Sunscreen Issues: 2007 Food and Drug Administration Sunscreen Labelling Recommendations and Combination Sunscreen/Insect Repellent Products. J. Am. Acad. Dermatol..

[44] Hamant E.S., Adams B.B. (2005). Sunscreen Use among Collegiate Athletes. J. Am. Acad. Dermatol..

[45] de Castro Maqueda G., Gutiérrez-Manzanedo J.V., González-Montesinos J.L., Vaz Pardal C., Rivas Ruiz F., de Troya Martín M. (2022). Sun Exposure and Photoprotection: Habits, Knowledge and Attitudes Among Elite Kitesurfers. J. Cancer Educ..

[46] De Castro-Maqueda G., Gutierrez-Manzanedo J.V., Lagares-Franco C., Linares-Barrios M., De Troya-Martin M. (2019). Photoprotection Practices, Knowledge and Sun-Related Skin Damage in Spanish Beach Handball Players. PeerJ.

[47] Navarrete-De Gálvez M., Ruiz Sánchez J.M., Navarrete-De Gálvez E., Aguilera J., Rivas-Ruiz F., de Troya-Martín M., Herrera-Ceballos E., de Gálvez M.V. (2022). Sun Exposure and Protection Habits in Transplant Athletes: An International Survey. Photodermatol. Photoimmunol. Photomed..

[48] Tenforde A.S., Fredericson M., Toth K.E.S., Sainani K.L. (2021). Sun Protective Behaviors and Attitudes of Runners. Sport.

[49] Gutiérrez-Manzanedo J.V., Vaz Pardal C., Blázquez-Sánchez N., De Gálvez M.V., Aguilera-Arjona J., González-Montesinos J.L., Rivas Ruiz F., De Troya-Martín M. (2022). Ultraviolet Exposure of Competitors during a Tokyo Olympic Sailing Regatta Test Event. Photodermatol. Photoimmunol. Photomed..

[50] Wysong A., Gladstone H., Kim D., Lingala B., Copeland J., Tang J.Y. (2012). Sunscreen Use in NCAA Collegiate Athletes: Identifying Targets for Intervention and Barriers to Use. Prev. Med..

[51] Buljan M., Kolić M., Šitum M., Šekerija M., Franceschi N. (2020). Do Athletes Practicing Outdoors Know and Care About the Importance of Photoprotection?. Acta Dermatovenerol. Croat..

[52] Fernández-Morano T., de Troya-Martín M., Rivas-Ruiz F., Fernández-Peñas P., Padilla-España L., Sánchez-Blázquez N., Buendía-Eisman A. (2017). Sun Exposure Habits and Sun Protection Practices of Skaters. J. Cancer Educ..

[53] Weikert A.E., Pagoto S.L., Handley E., Courtney J.B., Brunke-Reese D., Conroy D.E. (2021). Golfers’ Interest in Multilevel Sun-Protection Strategies. Int. J. Environ. Res. Public Health.

[54] Duarte A.F., Nagore E., Silva J.N.M., Picoto A., Pereira A.C., Correia O.J.C. (2018). Sun Protection Behaviour and Skin Cancer Literacy among Outdoor Runners. Eur. J. Dermatol..

[55] García-Malinis A.J., Gracia-Cazaña T., Zazo M., Aguilera J., Rivas-Ruiz F., de Troya Martín M., Gilaberte Y. (2021). Sun Protection Behaviors and Knowledge in Mountain Marathon Runners and Risk Factors for Sunburn. Actas Dermosifiliogr..

[56] Doncel Molinero D., Ruiz Paulano M., Rivas Ruiz F., Blázquez Sánchez N., de Gálvez Aranda M.V., de Castro Maqueda G., de Troya Martín M. (2022). Sun Protection Behaviour and Sunburns in Spanish Cyclists. J. Cancer Educ..

[57] Cohen P.H., Tsai H., Puffer J.C. (2006). Sun-Protective Behavior among High-School and Collegiate Athletes in Los Angeles, CA. Clin. J. Sport Med..

[58] Gutiérrez-Manzanedo J.V., De Castro-Maqueda G., Caraballo Vidal I., González-Montesinos J.L., Vaz Pardal C., Rivas Ruiz F., De Troya-Martín M. (2021). Sun-Related Behaviors, Attitudes and Knowledge among Paralympic Sailors. Disabil. Health J..

[59] Christoph S., Cazzaniga S., Hunger R.E., Naldi L., Borradori L., Oberholzer P.A. (2016). Ultraviolet Radiation Protection and Skin Cancer Awareness in Recreational Athletes: A Survey among Participants in a Running Event. Swiss Med. Wkly..

[60] Walkosz B., Voeks J., Andersen P., Scott M., Buller D., Cutter G., Dignan M. (2007). Randomized Trial on Sun Safety Education at Ski and Snowboard Schools in Western North America. Pediatr. Dermatol..

[61] Walkosz B.J., Buller D.B., Andersen P.A., Scott M.D., Dignan M.B., Cutter G.R., Maloy J.A. (2008). Increasing Sun Protection in Winter Outdoor Recreation: A Theory-Based Health Communication Program. Am. J. Prev. Med..

[62] Ally M.S., Swetter S.M., Hirotsu K.E., Gordon J., Kim D., Wysong A., Donnelly L., Li S., Nord K.M. (2018). Promoting Sunscreen Use and Sun-Protective Practices in NCAA Athletes: Impact of SUNSPORT Educational Intervention for Student-Athletes, Athletic Trainers, and Coaches. J. Am. Acad. Dermatol..

